# Model choice for estimating the association between exposure to chemical mixtures and health outcomes: A simulation study

**DOI:** 10.1371/journal.pone.0249236

**Published:** 2021-03-25

**Authors:** Lauren Hoskovec, Wande Benka-Coker, Rachel Severson, Sheryl Magzamen, Ander Wilson

**Affiliations:** 1 Department of Statistics, Colorado State University, Fort Collins, CO, United states of America; 2 Department of Environmental and Radiological Health Sciences, Colorado State University, Fort Collins, CO, United states of America; Stony Brook University, Graduate Program in Public Health, UNITED STATES

## Abstract

Challenges arise in researching health effects associated with chemical mixtures. Several methods have recently been proposed for estimating the association between health outcomes and exposure to chemical mixtures, but a formal simulation study comparing broad-ranging methods is lacking. We select five recently developed methods and evaluate their performance in estimating the exposure-response function, identifying active mixture components, and identifying interactions in a simulation study. Bayesian kernel machine regression (BKMR) and nonparametric Bayes shrinkage (NPB) were top-performing methods in our simulation study. BKMR and NPB outperformed other contemporary methods and traditional linear models in estimating the exposure-response function and identifying active mixture components. BKMR and NPB produced similar results in a data analysis of the effects of multipollutant exposure on lung function in children with asthma.

## Introduction

Individuals are continuously exposed to complex mixtures of environmental chemicals. Mounting evidence from epidemiological studies links environmental exposures to increased morbidity and mortality [1–5]. Traditional epidemiological studies have focused on a single pollutant and additive models with a small number of exposures; however, studying pollutants in isolation can lead to biased estimates [6, 7] and does not reflect the reality that people are jointly exposed to mixtures of pollutants. Hence, interest is rapidly growing in studying health outcomes associated with simultaneous exposure to mixtures of pollutants [8, 9]. The National Institute for Environmental Health Sciences (NIEHS) identified the study of mixtures as a goal in its 2012-2017 strategic plan while noting that this will require novel quantitative approaches [10]. As such, numerous statistical methods have been proposed. There is a need to identify the most appropriate statistical methods currently available for estimating health outcomes associated with exposure to mixtures [11, 12].

Studying health outcomes associated with exposure to mixtures is complicated by small effect sizes, highly correlated exposures, possible nonlinear and interaction effects, and often small sample sizes. In this context, traditional regression methods are often inadequate as they may yield biased or unstable estimates [13] and have low power to detect effects, especially in the case of nonlinear associations and interactions. Common methods designed for variable selection tend to incorrectly select predictors when many predictors are highly correlated [14] and classical model selection techniques ignore uncertainty in both the selected model and selected mixture components when estimating the exposure-response function [15, 16].

In a broad literature review, Davalos et al. [17] identified five classes of methods currently used in mixtures analyses: additive main effects (AME), effect measure modification (EMM), unsupervised dimension reduction (UDR), supervised dimension reduction (SDR), and nonparametric (NP). AME and EMM methods are typically regression based. AME allows only additive effects, while EMM includes multiplicative interactions. Hierarchical and penalized regression methods have been applied to AME and EMM models to identify important mixture components and improve precision [18–22]. The next two groups are dimension reduction techniques (UDR and SDR) that transform exposure data to reduce the dimension of the predictor and, therefore, the required parameter space. UDR methods such as *k*-means [23, 24] transform exposure data without regard to the health outcome [25–28]. SDR methods, including supervised principle components analysis [29], let the outcome inform exposure data transformation [30–35]. Finally, NP methods like Bayesian kernel machine regression [36] are flexible data-driven techniques for estimating a complex exposure-response function that may include interactions and nonlinear effects [37, 38].

Choosing an appropriate statistical model depends on the research objectives [11, 39] and requires understanding the empirical performance of methods. Recent studies have compared several methods in subsets of the model classes proposed. Among those evaluated include linear regression AME [40] and EMM methods [14], principle components analysis [34], structural equation models [41], Bayesian kernel machine regression [41], and Bayesian semiparametric regression [42]. These studies highlight challenges induced by highly correlated data in estimating complex exposure-response functions and characterizing uncertainty. To our knowledge, there has been no formal evaluation of methods from all five classes identified by Davalos et al. [17] in a single simulation study.

Evaluating the empirical performance of methods across a wide spectrum of model classes is important as it guides researchers in choosing across classes of models and aids in interpreting results and understanding the limitations of epidemiological studies using these methods. In addition, the existing literature is sparse with regards to a comparison among Bayesian methods, which are favorable in the multipollutant setting as they can incorporate prior information and fully characterize uncertainty [12, 34, 43]. To this end, we focus on a comparison of Bayesian methods across a variety of model classes in this paper. By comparing performance across classes of models, researchers can also gain insight into promising future directions for statistical methods development.

Motivated by research linking mixtures of air pollutant and pesticide exposures to child respiratory health, we conducted a simulation study to compare contemporary methods developed for estimating the association between health outcomes and exposure to mixtures. We considered one method from each of the five classes identified by Davalos et al. [17] and evaluated each method in three data-generating scenarios. The data-generating scenarios cover a range of linear to nonlinear functions of multiple pollutants with synergistic effects on the response in order to test each method in its ability to estimate both simple and complex exposure-response functions that may be encountered in practice.

In contrast to many recent studies that have compared methods from a conceptual standpoint or compared their performance in the analysis of a single data set, the primary contribution of our work is to compare diverse methods in a simulation study addressing a variety of research questions. Specifically, we quantified four aspects of model performance corresponding to previously identified epidemiological questions of interest: 1) how well does the model estimate the exposure-response function, 2) can the model identify important mixture components, 3) can the model identify components not associated with the outcome, and 4) can the model identify interactions among exposures [39].

A secondary contribution of our work is to provide software for the tested methods that currently lack software. Our simulation study describes the strengths and weaknesses of each method and available software encourages practitioners to use the most appropriate methods in a given application. Software is available in the form of the R package mmpack [44] to reproduce the simulation. Further, the software allows researchers to easily conduct a simulation study using the same methods and simulated exposure-response functions but substituting in their own exposure data which will have a different correlation structure and may result in different model performance. Hence, researchers can determine which methods are most appropriate for their own study. Finally, we applied each method to a data analysis of a cohort study investigating the relationship between air pollutant and pesticide exposures and lung function in children with asthma. We describe the differences in results among the methods, highlighting the importance of model choice.

## Materials and methods

### Data

#### Health data

This study was approved by the Institutional Review Board of Colorado State University, Protocol Number 19-9437H. This was a secondary data analysis from a closed cohort with all personal identifying information stripped from the database. We used data from Fresno Asthmatic Children’s Environment Study (FACES). The study design, including recruitment, eligibility criteria, and measurement procedures, is described elsewhere [45–50]. FACES includes data for children aged 6-11 years with asthma symptoms at the time of enrollment and living within a 20 kilometer radius of one of Fresno‘s EPA air quality monitoring sites. The health outcome of interest was baseline forced expiratory volume in the first second (FEV_1_) measured via spirometry. We regressed FEV_1_ on age, sex, height and ethnicity and used the residuals as the outcome in our data analysis [51–53]. Age, sex, height, and ethnicity are well-known predictors of FEV_1_ so we remove all variation from these predictors before looking into the effects of air pollution and pesticide exposure on FEV_1_. Other covariates have not been as well studied regarding their association with FEV_1_ and are including the model as potential confounding variables. Complete exposure, health, and covariate data were available for 153 children.

The data contain information on covariates and potential confounding variables (S1 Table in S1 File). We included average temperature and precipitation over three months, the temporal scale of the pesticide exposure data, prior to baseline as covariates. Subject-specific covariates include body mass index (BMI, kg/m^2^) and indicators for: self-reported residence within one block of a freeway, any smoking in the home, positive atopy or allergy test, modified Global Initiative for Asthma (GINA) score ≥ 3 at baseline, household income greater than $30K/year, mother having post-secondary education, child not covered by insurance, and season of baseline spirometry test. Temperature, precipitation, and BMI were scaled to have mean 0 and variance 1. Approximately 1% of the covariate data was missing, including any smoking in the home (16%), household income (3%), and mother having post-secondary education (1%). As all covariates with missing data were binary variables, we singly imputed the missing values with 0 and then added a dummy variable for each covariate with any missing data that indicated which values of that covariate were missing.

#### Air pollution and pesticide data

We obtained air pollution data from the EPA Air Quality System Data Mart. Air pollutant concentrations were calculated as 24-hour averages for particles ≤ 2.5 *μ*m in aerodynamic diameter (PM_2.5_) and particles ≤ 10 *μ*m in aerodynamic diameter (PM_10_), 8-hour daily maximum levels for ozone (O_3_) and one-hour daily maximum levels for nitrogen dioxide (NO_2_) [47]. Concentrations were taken from the air monitoring site closest to each child’s residence and exposure levels were summarized as averages over three months prior to baseline spirometry tests to be consistent with available pesticide exposure data. Due to right-skewed distributions, air pollutant exposures were square-root transformed and then scaled to have mean 0 and variance 1.

We obtained data on the date, location, and amount (kilograms) of applied agricultural pesticides from the California Pesticide Use Report (PUR) [54]. Based on previous evidence linking pesticide exposure to respiratory illness [55, 56], we considered three pesticide classes: carbamates (C), methyl bromide (MeBr), and organophosphates (OP). Pesticide exposures were estimated using the purexposure [57] package in R. We applied inverse distance weighting to the total reported pesticide amount over three months prior to baseline spirometry tests (as PUR reports are aggregated quarterly) to estimate exposures within a 3km buffer of each child’s residence. Pesticide exposures were also highly skewed and so were square-root transformed and then scaled to have mean 0 and variance 1.

Exposure data summary statistics are shown in Table 1. Strong Spearman correlation existed between NO_2_ and PM_2.5_ (*ρ* = 0.88) and between NO_2_ and PM_10_ (*ρ* = 0.72). Moderate Spearman correlation existed between PM_2.5_ and PM_10_ (*ρ* = 0.67), O_3_ and NO_2_ (*ρ* = -0.63), O_3_ and PM_2.5_ (*ρ* = -0.54), O_3_ and OP (*ρ* = 0.53), and OP and NO_2_ (*ρ* = -0.53) (Table 2).

**Table 1:** Pesticide and air pollutant exposure data summary statistics. Table shows mean, standard deviation (SD), minimum, 25th percentile, median, 75th percentile, and maximum concentration for each exposure.

	mean	SD	min	25^th^	median	75^th^	max
C × 10^6^ (kg/3km^2^)	0.15	0.33	0.00	0.00	0.00	0.15	2.35
MeBr × 10^6^ (kg/3km^2^)	3.88	9.90	0.00	0.00	0.00	0.00	48.92
OP × 10^6^ (kg/3km^2^)	0.93	1.08	0.00	0.00	1.11	1.17	5.40
O_3_ (ppb)	0.04	0.01	0.01	0.03	0.04	0.04	0.06
NO_2_ (ppb)	15.48	3.26	9.49	12.64	14.42	17.96	23.07
PM_2.5_ (*μ*g/m^3^)	16.35	9.80	6.66	10.14	11.23	18.20	40.21
PM_10_ (*μ*g/m^3^)	37.89	10.68	19.55	30.30	32.49	47.23	65.94

**Table 2:** Spearman correlation among all pairs of air pollutant and pesticide exposures.

	MeBr	OP	O_3_	NO_2_	PM_2.5_	PM_10_
C	0.27	0.12	0.09	0.08	0.06	0.01
MeBr		-0.08	0.02	0.07	-0.03	-0.13
OP			0.53	-0.53	-0.38	-0.24
O_3_				-0.63	-0.54	-0.22
NO_2_					0.88	0.72
PM_2.5_						0.67

### Statistical methods

Our primary interest was to estimate the association between exposures to *p* pollutants **x**_*i*_ = (*x*_*i*1_, …, *x*_*ip*_)^*T*^ and a continuous outcome *y*_*i*_, while controlling for *q* potential confounders **w**_*i*_ = (*w*_*i*1_, …, *w*_*iq*_)^*T*^ in a sample *i* = 1, …, *n*. We considered five recently proposed methods. The first two are the AME model nonparametric Bayes shrinkage with main effects only (NPBr) and the EMM model nonparametric Bayes shrinkage with main effects and all pairwise multiplicative interactions (NPB) as proposed by Herring [19]. The next two models are unsupervised (UPR) and supervised Bayesian profile regression (SPR) as proposed by Molitor et al. [58]. The fifth is the NP model Bayesian kernel machine regression (BKMR) [36]. We chose these methods since they represent the five classes identified by Davalos et al. [17] and are recently developed Bayesian methods for estimating health outcomes associated with exposure to mixtures. These five methods cover a variety of exposure-response function shapes, handle multicollinearity in various ways, and include options for variable selection. BKMR is presented exactly as proposed by Bobb et al. [36]; NPB and SPR have been modified to accommodate the continuous outcome with normal residuals rather than the logistic model originally proposed by Herring [19] and Molitor et al. [58], respectively; and NPBr and UPR are further modifications of those previously introduced methods. For a baseline comparison, we also included a normal linear model with main effects only (LM) and with all pairwise interactions (LM-int), both estimated with least squares. All models considered in this paper have the form

$$\begin{array}{c} y_{i}=h\left(x_{i}\right)+w_{i}^{T}\gamma +\epsilon_{i}, \end{array}$$ (1)

where *ϵ*_*i*_ are independent N(0, *σ*^2^) and *h*(**x**_*i*_) represents the exposure-response function. All models were fit in R version 3.6.0 [59].

#### Nonparametric Bayes shrinkage

Nonparametric Bayes shrinkage [19] was originally introduced as a logistic regression EMM model and was adapted to the linear regression setting used here. We consider two variations. NPB, originally proposed by Herring [19] is an EMM model including main effects and all pairwise interactions, where

$$\begin{array}{c} h\left(x_{i}\right)=\sum_{j=1}^{p}x_{ij}\beta_{j}+\sum_{j=1}^{p-1}\sum_{k=j+1}^{p}x_{ij}x_{ik}\zeta_{jk}. \end{array}$$ (2)

NPBr is a reduced AME model not originally proposed in Herring [19] that includes only main effects:

$$\begin{array}{c} h\left(x_{i}\right)=\sum_{j=1}^{p}x_{ij}\beta_{j}. \end{array}$$ (3)

Both models place a Dirichlet Process (DP) prior on regression coefficients. The base distribution of the DP is a finite mixture of a normal distribution and a point mass at 0 to induce sparsity in the model. Hence, some coefficients are set exactly to 0, effectively selecting out variables that do not contribute to the health outcome. Correlated exposures can be clustered and assigned equal regression coefficients to reduce variance [19, 60]. This effectively reparameterizes the model to have a single effect for the sum of two correlated predictors and is particularly advantageous in situations where it is difficult to differentiate the effects of two highly correlated predictors. The DP prior for main effects is constructed as:

$$\begin{array}{ccc} \beta_{j}|D_{1} & ∼ & D_{1},j=1,\ldots p \end{array}$$ (4)

$$\begin{array}{ccc} D_{1}|\alpha_{1},D_{01} & ∼ & \text{DP}(\alpha_{1}D_{01}) \end{array}$$

$$\begin{array}{ccc} D_{01}|\pi_{01},G_{1} & = & \pi_{01}\delta_{0}+\left(1-\pi_{01}\right)G_{1} \end{array}$$

$$\begin{array}{ccc} G_{1}|\mu_{1},\phi_{1}^{2} & \equiv & \text{N}(\mu_{1},\phi_{1}^{2}), \end{array}$$

where *δ*_0_ represents the Dirac delta function at 0. The model is completed with standard hyperpriors *α*_1_ ∼ Gamma(*α*_*α*1_, *β*_*α*1_), *π*_01_ ∼ Beta(*α*_*π*1_, *β*_*π*1_), $\mu_{1}∼\text{N}\left(0,\sigma_{\mu 1}^{2}\right)$, and $\phi_{1}^{-2}∼\text{Gamma}\left(\alpha_{\phi 1},\beta_{\phi 1}\right)$. The DP prior for interactions is similarly constructed. Specifically,

$$\begin{array}{ccc} \zeta_{jk}|D_{2} & ∼ & D_{2},j=1,\ldots p-1\;\&\;k=j+1,\ldots ,p \end{array}$$ (5)

$$\begin{array}{ccc} D_{2}|\alpha_{2},D_{02} & ∼ & \text{DP}(\alpha_{2}D_{02}) \end{array}$$

$$\begin{array}{ccc} D_{02}|\pi_{02},G_{2} & = & \pi_{02}\delta_{0}+\left(1-\pi_{02}\right)G_{2} \end{array}$$

$$\begin{array}{ccc} G_{2}|\mu_{2},\phi_{2}^{2} & \equiv & \text{N}(\mu_{2},\phi_{2}^{2}). \end{array}$$

The hyperpriors on *α*_2_, *π*_02_, *μ*_2_, and $\phi_{2}^{-2}$ come from the same families specified for the main effects. The distributions on the main effects and interactions are independent a priori.

Posterior inclusion probabilities (PIPs) are calculated for each mixture component as the posterior probability of the regression coefficient being assigned a non-zero value. Both NPBr and NPB were fit using the R package mmpack [44].

#### Bayesian profile regression

Bayesian profile regression is a dimension reduction approach that classifies pollutant exposure profiles, **x**_*i*_, into a parsimonious set of clusters using a DP mixture model (DPMM) [58, 61]. Each cluster represents a group of observations with similar exposure levels across the vector of pollutants. The health outcome is regressed on cluster indicators to estimate

$$\begin{array}{c} h\left(x_{i}\right)=\theta_{c} \end{array}$$ (6)

if profile **x**_*i*_ is assigned to cluster *c*. We introduce a latent variable *z*_*i*_ = *c* if exposure profile *i* is assigned to cluster *c*. Conditional on cluster assignment, the model for an individual exposure profile is

$$\begin{array}{ccc} x_{i}|z_{i}=c,\mu_{c},\Sigma_{c} & ∼ & \text{N}(\mu_{c},\Sigma_{c}) \end{array}$$ (7)

$$\begin{array}{ccc} \mu_{c} & ∼ & \text{N}(\nu_{0},\Lambda_{0}) \end{array}$$

$$\begin{array}{ccc} \Sigma_{c}^{-1} & ∼ & {\text{Wish}}_{\text{p}}\left(R,r\right). \end{array}$$

The DPMM for cluster assignment places a truncated stick-breaking prior on the assignment probabilities to each cluster. The stick-breaking process and cluster assignment model are

$$\begin{array}{ccc} V_{1},\ldots ,V_{C-1}|\alpha & ∼ & \text{Beta}\left(1,\alpha \right),\;V_{C}=1 \end{array}$$ (8)

$$\begin{array}{ccc} \alpha & ∼ & \text{Gamma}(\alpha_{\alpha },\beta_{\alpha }) \end{array}$$

$$\begin{array}{ccc} P\left(z_{i}=c\right)=\psi_{c} & = & V_{c}\prod_{h=1}^{c-1}\left(1-V_{h}\right) \end{array}$$

$$\begin{array}{ccc} z_{i} & ∼ & \text{Categorical}(\psi ). \end{array}$$

Subject to a maximum of *C* clusters, the DPMM allows the number of non-empty clusters to be estimated from the data. To identify the most optimal partitioning of the data, we follow the approach described in Dahl [62] and Molitor et al [58]. First, we construct an *n* × *n* score matrix at each iteration with a 1 in the *i*, *j* location if individuals *i* and *j* belong to the same cluster and a 0 otherwise. Then we calculate a probability matrix **S** by averaging the score matrices. The most optimal clustering is the clustering from the MCMC iteration that has a score matrix with minimum least squared distance to the probability matrix **S**. We calculate model averaged estimates of the cluster-specific parameters *θ*_*c*_ to incorporate the uncertainty present in the best clustering [58].

The model has been extended to include variable selection to identify mixture components actively contributing to cluster assignment [63–65]. Briefly, binary random variables are introduced that indicate whether the mean for a mixture component within a cluster is unique to that cluster or common among all clusters. Hence, mixture components that are selected into the model are interpreted as being informative in partitioning the exposure data into clusters, but are not necessarily related to the health outcome.

We consider two variations of profile regression. The first, supervised profile regression (SPR), originally introduced by Molitor et al. [58] belongs to the SDR class of methods since cluster assignments are influenced by the health outcome. The second is an unsupervised adaptation (UPR) not originally proposed by Molitor et al. [58] that belongs to the UDR class. The difference between the two variations manifests when the latent cluster assignment variable *z*_*i*_ is updated. In the supervised case, we jointly model the response and estimate cluster assignments. Hence, there is feedback between the health outcome model and the profile assignment model where the health outcomes can influence cluster assignment. The full conditional for *z*_*i*_ depends on both the likelihood of exposures **x**_*i*_ and the likelihood of the response *y*_*i*_:

$$\begin{array}{ccc} P(z_{i}=c|x_{i},y_{i},\cdot ) & = & \frac{\psi_{c}f\left(x_{i}|z_{i}=c,\mu_{c},\Sigma_{c}\right)f\left(y_{i}|z_{i}=c,\theta_{c},\beta ,\sigma^{2}\right)}{\sum_{c=1}^{C}\psi_{c}f\left(x_{i}|z_{i}=c,\mu_{c},\Sigma_{c}\right)f\left(y_{i}|z_{i}=c,\theta_{c},\beta ,\sigma^{2}\right)}. \end{array}$$ (9)

Hence, in SPR, individuals with similar exposure profiles but different health outcomes may be assigned to different clusters depending on their responses.

The unsupervised case involves a two-step procedure where we first estimate cluster assignments independently of the response and then model the response conditional on cluster assignment. Here, *z*_*i*_ depends only on the exposure likelihood:

$$\begin{array}{ccc} P(z_{i}=c|x_{i},\cdot ) & = & \frac{\psi_{c}f\left(x_{i}|z_{i}=c,\mu_{c},\Sigma_{c}\right)}{\sum_{c=1}^{C}\psi_{c}f\left(x_{i}|z_{i}=c,\mu_{c},\Sigma_{c}\right)}. \end{array}$$ (10)

Since the response does not inform cluster assignment in UPR, there may be high uncertainty in the estimates of the cluster indicators *θ*_*c*_ if individuals with similar exposure profiles have very different health outcomes.

We fit SPR using the R package PReMiuM [65] and UPR using the R package mmpack developed for this paper [44].

#### Bayesian kernel machine regression

Bayesian kernel machine regression (BKMR) [36] belongs to the NP class of methods and flexibly models the exposure-response function to allow for nonlinear associations and higher order interactions. In BKMR, *h*(**x**) is a smooth function represented using a Gaussian kernel. The response is modeled as

$$\begin{array}{ccc} y_{i} & ∼ & N(h_{i}+w_{i}^{T}\gamma ,\sigma^{2}) \end{array}$$ (11)

$$\begin{array}{ccc} h\equiv {\left(h_{1},\ldots ,h_{n}\right)}^{T} & ∼ & N(0,\tau K), \end{array}$$

where *K* is the kernel matrix with (*i*, *i*′) element $K\left(x_{i},x_{i^{\prime }}\right)=\exp\left\{-\sum_{j=1}^{p}r_{j}{\left(x_{ij}-x_{i^{\prime }j}\right)}^{2}\right\}$, *τ* is a hyperparameter, and **r** = (*r*_1_, …, *r*_*p*_)^*T*^ are variable selection parameters. Estimated health outcomes for individuals with similar exposure levels across the *p* predictors are shrunk towards each other, resulting in a smooth but flexible exposure-response function.

BKMR allows for both component-wise and hierarchical variable selection (HVS) to identify important mixture components. In our simulation and data analysis, we implemented component-wise variable selection and calculated PIPs for each exposure. We also implemented HVS in our data analysis to address sensitivity of results. We partitioned the mixture components into groups of air pollutants (PM_2.5_, PM_10_, NO_2_, and O_3_) and pesticides (C, MeBr, and OP) and calculated PIPs for each group (group PIPs) and each component within a group, conditional on group inclusion (conditional PIPs). We fit BKMR using the R package bkmr [66].

### Simulation study design

We evaluated the proposed methods in a simulation study. We ensure a realistic correlation structure among the pollutants by using the observed exposure data from 153 individuals in the FACES data set in our simulation study. We also use the observed covariate data in our simulation study. Health responses were simulated for three exposure-response scenarios, denoted *h*_*k*_, *k* = 1, 2, 3, as $y_{i}=h_{k}\left(x_{i}\right)+w_{i}^{T}\gamma +\varepsilon_{i}$, with *ε*_*i*_ ∼ N(0, 1). The covariate coefficients *γ*_1_, …, *γ*_*q*_ were simulated as independent N(0, 1).

The first scenario, *h*_1_ (linear), is an EMM model. For exposures *x*_*j*_, *j* = 1, …, 4, the exposure-response function is

$$\begin{array}{c} h_{1}\left(x\right)=x_{1}-x_{2}+x_{3}-x_{4}+0.7x_{1}x_{2}-0.5x_{3}x_{4}. \end{array}$$ (12)

Second, *h*_2_ (nonlinear) includes nonlinear sigmoidal functions of three pollutants and a multiplicative interaction between two of those pollutants:

$$\begin{array}{c} h_{2}\left(x\right)=\frac{2}{1+\exp(-3x_{1})}+\frac{2}{1+\exp(-5x_{2})}-\frac{2}{1+\exp(-5x_{3})}-0.4x_{1}x_{2}. \end{array}$$ (13)

Last, *h*_3_ (fixed profiles) groups individuals into four distinct clusters based on dichotomous cut-offs for two pollutants. We assign a constant health effect to individuals in the same cluster:

$$\begin{array}{c} h_{3}\left(x\right)=\{\begin{array}{cc} -2, & x_{1}\leq \text{median}\left(x_{1}\right)\;\text{and}\;x_{2}\leq \text{median}\left(x_{2}\right)\\ -1, & x_{1}\leq \text{median}\left(x_{1}\right)\;\text{and}\;x_{2}>\text{median}\left(x_{2}\right)\\ 0, & x_{1}>\text{median}\left(x_{1}\right)\;\text{and}\;x_{2}\leq \text{median}\left(x_{2}\right)\\ 2, & x_{1}>\text{median}\left(x_{1}\right)\;\text{and}\;x_{2}>\text{median}\left(x_{2}\right). \end{array} \end{array}$$ (14)

We selected these three exposure-response scenarios to cater to different methods in our simulation study. The linear scenario plays to NPBr and NPB, the nonlinear scenario plays to BKMR, and the fixed profiles scenario plays to UPR and SPR. We hypothesize that the methods to which each scenario caters will perform best in that scenario. We are interested in evaluating how methods perform in exposure-response scenarios for which they were not explicitly developed.

We simulated 200 data sets for each scenario and fit all five Bayesian methods plus LM and LM-int. As results can be sensitive to which pollutants, *x*_*j*_, *j* = 1, …, 4, are included in *h*(**x**), we randomly selected pollutants to be the active components in each simulated data set. All pollutants, even those not selected as one of the active components, are included as inputs in the estimated models. By randomly selecting which exposures are the active components of the mixture, each simulated data set has a different correlation structure among the active exposures, which adds robustness to our simulation study results. We calculated the Calinski-Harabasz index [67], the silhouette statistic [68], and the number of clusters to maximize the gap width [69] to measure the grouping structure of the data generated by the fixed profiles scenario. Although the exposure data remains the same for each data set, the exposures used in the exposure-response function differ for each data set; hence the clustering in the fixed profiles scenario, which is based on the response, differs for each data set. Across the 200 data sets used in our simulation study, the median Calinski-Harabasz index was 22.54, the median silhouette was 0.15, and the median number of clusters to maximize the gap width was 6. The distribution of each of these statistics can be found in S2 Table in S1 File. In general, the fixed profiles scenario did not always generate a strong grouping structure with this data, but instead represents a wide variety of clustering schemes.

We evaluated exposure-response function estimation using root mean squared error (RMSE) and interval coverage (Cvg). RMSE was calculated as $\sqrt{\frac{1}{n}\sum_{i=1}^{n}{\left[h\left(x_{i}\right)-\hat{h}\left(x_{i}\right)\right]}^{2}}$ and coverage was calculated as the percent of *h*(**x**_*i*_)’s covered by 95% credible or confidence intervals. RMSE measures the variation between estimated and true values of the exposure-response function. Coverage measures how often the 95% credible or confidence intervals for the estimated exposure-response function capture the true mixture effect. A method with high RMSE and low coverage fails to capture the overall mixture effect. In this way, RMSE and coverage measure the ability of each method to capture the overall mixture effect.

We summarized variable selection through true and false selection rates. In the Bayesian methods, we consider a variable with a PIP above 0.5 as selected into the model [70]. In LM and LM-int, a variable is selected if the 95% confidence interval for the respective regression coefficient does not contain 0. We calculated true selection rate (TSR) as the proportion of mixture components active in the exposure-response function as main effects that were selected into the model as main effects, and false selection rate (FSR) as the proportion of mixture components not in the exposure-response function as main effects that were selected into the model as main effects. All seven exposures are included in the models as inputs, but the active mixture components are those that define the exposure-response function for each simulated data set. For scenario 1, the active main effects are the randomly selected exposures denoted by *x*_1_, *x*_2_, *x*_3_, and *x*_4_; for scenario 2, the active main effects are *x*_1_, *x*_2_, and *x*_3_; and for scenario 3 the active main effects are *x*_1_ and *x*_2_. In most methods (NPBr, UPR, SPR, BKMR, and LM), TSR and FSR are calculated only for main effects. In NPB we can calculate PIPs for interactions and in LM-int we can calculate confidence intervals for the interaction effects. Hence, we also evaluate variable selection rates for interactions in NPB and LM-int. We calculate true selection rate for interactions (TSR_int_) as the proportion of the exact pairwise interactions active in the exposure response function that were selected into the model as interactions, and false selection rate for interactions (FSR_int_) as the proportion of interactions that were not active in the exposure-response function that were selected into the model as interactions. In scenario 1, the true active interactions are *x*_1_ *x*_2_ and *x*_3_ *x*_4_ and in scenarios 2 and 3 the only active interaction is *x*_1_ *x*_2_.

We assessed convergence for a few simulated data sets by visualizing trace plots and comparing results from multiple chains. We found evidence of convergence by 20,000 iterations for all methods. To ensure convergence across all simulated data sets, we based inference on 25,000 samples after a burn-in of 25,000 samples.

We conducted three additional simulation studies to further assess method performance. First, we considered a null scenario, *h*_4_(**x**), where none of the exposures are associated with the response. Second, we considered a complex mixture scenario, *h*_5_(**x**), where we simulated data for seven additional pollutants to have a total of 14 mixture components. Third, we applied our original simulation study design to a larger sample of size *n* = 1000 for each data set. Details on the additional simulations can be found in S1 Appendix in S1 File.

### Data analysis

We conducted a data analysis on 153 individuals with complete data in the FACES data set. We used regression-adjusted FEV_1_ as the outcome. S1 Table in S1 File summarizes the characteristics of the sample. Pesticide and air pollutant exposures and covariate data were identical to that in our simulation study (Tables 1, 2, and S1 Table in S1 File). We fit the same models as in the simulation study. Prior specification for the Bayesian models is listed in S2 Appendix in S1 File.

## Results

### Simulation study results

Simulation results are shown in Table 3. Standard errors are shown in S3–S5 Tables in S1 File. We show the computational time for each method to run for 5000 iterations in Table 4.

**Table 3:** Summary of method performance in three data-generating scenarios. Table shows means across all data sets for: root mean squared error (RMSE), coverage (Cvg), true selection rate for main effects (TSR), false selection rate for main effects (FSR), true selection rate for interactions (TSR_int_), and false selection rate for interactions (FSR_int_). Top-performing methods will have low RSME, coverage near the nominal level (0.95), high TSR and low FSR. For each measure and exposure-response scenario, results from the top-performing method(s) are listed in bold.

Method	RMSE	Cvg	TSR	FSR	TSR_int_	FSR_int_
*h*_1_(**x**): linear with multiplicative interactions						
NPBr	1.02	0.73	0.85	0.35	–	–
NPB	**0.54**	**0.95**	0.92	0.10	**0.59**	**0.02**
UPR	2.01	0.56	0.25	0.26	–	–
SPR	1.59	0.54	0.63	0.53	–	–
BKMR	**0.55**	**0.96**	**1.00**	0.39	–	–
LM	1.01	0.73	0.84	0.29	–	–
LM-int	0.73	**0.95**	0.68	**0.04**	0.32	0.04
*h*_2_(**x**): nonlinear with multiplicative interactions						
NPBr	0.77	0.80	0.79	0.22	–	–
NPB	0.69	0.86	0.78	0.16	**0.25**	**0.01**
UPR	1.42	0.56	0.27	0.24	–	–
SPR	1.27	0.58	0.68	0.58	–	–
BKMR	**0.59**	**0.92**	**0.96**	0.48	–	–
LM	0.78	0.81	0.78	0.17	–	–
LM-int	0.89	**0.91**	0.54	**0.08**	0.20	0.07
*h*_3_(**x**): constant function of fixed profiles						
NPBr	1.11	0.66	0.66	**0.11**	–	–
NPB	1.02	0.75	0.68	0.13	0.06	**0.02**
UPR	1.41	0.55	0.27	0.25	–	–
SPR	1.38	0.54	0.68	0.59	–	–
BKMR	**0.69**	**0.91**	**0.97**	0.64	–	–
LM	1.13	0.70	0.69	0.14	–	–
LM-int	0.99	**0.91**	0.56	0.14	**0.12**	0.11

**Table 4:** Computational time for each method to run 5000 iterations on MacBook Pro in R version 3.6.1. Time is reported in seconds. Results reflect 10 evaluations of each method.

method	minimum	mean	maximum
NPBr	6.90	7.03	7.17
NPB	24.73	24.95	25.23
BKMR	219.43	222.96	235.41
UPR	57.82	58.66	59.50
SPR	90.34	92.47	98.65

Overall BKMR and NPB were the best performing methods with BKMR performing slightly better in the nonlinear and fixed profiles scenarios. Regarding RMSE for the exposure-response function, BKMR (RMSE = 0.55) and NPB (RMSE = 0.54) tied for lowest in the linear scenario. In the nonlinear scenario, BKMR (RMSE = 0.59) pulled slightly ahead of NPB (RMSE = 0.69), while in the fixed profiles scenario, BKMR (RMSE = 0.69) outperformed all other methods by a substantial margin. UPR had the highest RMSE in all three scenarios with SPR having the second highest RMSE.

In addition to having the lowest RMSE in all three scenarios, BKMR consistently had interval coverage closest to the nominal level. LM-int also had interval coverage near the nominal level in all three scenarios and NPB performed well in the linear scenario. BKMR (Cvg = 0.96), NPB (Cvg = 0.95), and LM-int (Cvg = 0.95) all achieved the nominal coverage level (0.95) in the linear scenario. In the nonlinear scenario, BKMR (Cvg = 0.92) and LM-int (Cvg = 0.91) came closest to the nominal level, with NPB next best but trailing behind (Cvg = 0.86). BKMR (Cvg = 0.91) and LM-int (Cvg = 0.91) had the highest coverage by far in the fixed profiles scenario. Again, UPR and SPR performed poorly with the lowest coverage in all three scenarios.

The story is more complex when it comes to variable selection. While BKMR had the highest TSR in all three scenarios, it also had the highest FSR. Again, NPB performed very well in the linear scenario but not as well in the other scenarios, while UPR and SPR had consistently poor selection rates. Regarding TSR, BKMR (TSR = 1.00) and NPB (TSR = 0.92) performed best in the linear scenario. BKMR had the highest TSR in the nonlinear scenario (TSR = 0.96), where the next best methods, NPBr, NPB, and LM, all had mean TSR just under 0.80. BKMR is singled out with the best TSR in the fixed profiles scenario (TSR = 0.97). UPR, SPR, and LM-int tended to have low TSR in all three scenarios.

A low false selection rate indicates a model does not erroneously classify exposures as associated with the outcome when they are not. Here, BKMR had some of the highest FSR across the three scenarios. In the linear scenario, LM-int (FSR = 0.04) and NPB (FSR = 0.10) had the lowest FSR. LM-int also had the lowest FSR in the nonlinear scenario (FSR = 0.08). In the fixed profiles scenario, NPBr, NPB, LM, LM-int all had similar FSR at or below 0.14. Along with BKMR, SPR had high FSR in all three scenarios.

When considering overall variable selection performance, NPB takes the top spot in the linear scenario, with high TSR and low FSR. No method was able to simultaneously achieve dominant TSR and FSR in the nonlinear or fixed profiles scenarios.

Only NPB and LM-int directly parameterized variable selection for interactions in an easily interpretable manner. Interpretable variable selection for interactions is itself an advantage of these approaches over the other methods. In the linear scenario, NPB (TSR_int_ = 0.59) had higher TSR_int_ than LM-int (TSR_int_ = 0.32). Both methods had poor TSR_int_ in the nonlinear and fixed profiles scenarios, with values at or below 0.25. Regarding FSR_int_, both methods performed well in all three scenarios, with FSR_int_ consistently at or below 0.11.

The additional simulations produced similar results, with NPB and BKMR being consistently top-performing methods in terms of estimating the exposure-response function and identifying active mixture components. In the null scenario, NPBr and NPB had lowest FSR, meaning these methods were the best at not selecting any mixture components into the model when none were associated with the response (S6 Table in S1 File). Results from the complex mixture scenario largely mirrored those from the linear scenario (S7 Table in S1 File). BKMR and NPB remained top-performing in the larger sample size simulation and TSR improved for all methods. Here, UPR and SPR had high TSR and FSR, meaning they often selected all of the mixture components into the model (S8 Table in S1 File).

### Data analysis results

The results from our analysis of the FACES data set varied across the methods. First we consider the traditional models LM and LM-int. LM showed evidence for main effects of NO_2_ ($\hat{\beta }$: -0.32, CI: -0.54, -0.10) and PM_10_ ($\hat{\beta }$: 0.19, CI: 0.02, 0.35). LM-int showed evidence for main effects of MeBr ($\hat{\beta }$: 0.17, CI: 0.05, 0.29), NO_2_ ($\hat{\beta }$: -0.68, CI: -1.10, -0.25), and PM_10_ ($\hat{\beta }$: 0.50, CI: 0.08, 0.93) and an interaction between C and PM_2.5_ ($\hat{\beta }$: 0.28, CI: 0.01, 0.54) (Table 5). The results from the linear models indicating a protective effect of PM_10_ are counter-intuitive as there is an extensive literature on the deleterious health effects of PM on lung function. None of the other methods found evidence of protective effects for any of the exposures.

**Table 5:** Results from analysis of FACES data set using LM and LM-int. Table includes effect estimates ($\hat{\beta }$), 95% confidence intervals, and associated *p*-values for all main effects in LM and LM-int plus the interaction effects in LM-int with *p*-values ≤ 0.10. The regression coefficient $\hat{\beta }$ is the expected change in FEV_1_ for a 1 standard deviation increase in the square root transformed exposures.

	LM			LM-int		
	$\hat{\beta }$	95% CI	*p*-value	$\hat{\beta }$	95% CI	*p*-value
C	0.04	(-0.03, 0.11)	0.24	0.05	(-0.08, 0.19	0.44
MeBr	0.00	(-0.06, 0.07)	0.96	0.17	(0.05, 0.29)	0.01
OP	0.05	(-0.03, 0.13)	0.24	0.02	(-0.17, 0.22)	0.80
O_3_	-0.06	(-0.20, 0.07)	0.36	-0.13	(-0.32, 0.06)	0.17
NO_2_	-0.32	(-0.54, -0.10)	0.01	-0.68	(-1.10, -0.25)	0.00
PM_2.5_	-0.01	(-0.20, 0.17)	0.90	-0.11	(-0.48, 0.26)	0.55
PM_10_	0.19	(0.02, 0.35)	0.03	0.50	(0.08, 0.93)	0.02
C:PM_2.5_	–	–	–	0.28	(0.01, 0.54)	0.04
OP:PM_10_	–	–	–	0.31	(-0.01, 0.62)	0.05
NO_2_:PM_10_	–	–	–	0.33	(-0.05, 0.72)	0.09

Next we consider the five contemporary methods. NPBr did not identify any exposures with PIPs above 0.5. The exposure with the highest PIP was NO_2_ (PIP = 0.47), which was estimated to be negatively associated with FEV_1_ ($\hat{\beta }$: -.08, CI: -0.35, 0.00). In NPB, NO_2_ was selected (PIP = 0.60) and was also negatively associated with FEV_1_ ($\hat{\beta }$: -0.12, CI: -0.36, 0.00) (Table 6). No other main effects or interactions were selected by either method (S10 Table in S1 File).

**Table 6:** Results from analysis of FACES data set using NPBr and NPB. Table shows estimates ($\hat{\beta }$), 95% credible intervals, and posterior inclusion probabilities (PIP) for main effect exposures in NPB and NPBr. The regression coefficient $\hat{\beta }$ is the expected change in FEV_1_ for a 1 standard deviation increase in the square root transformed exposures. All interaction effects in NPB had posterior inclusion probabilities below 0.12.

	NPBr			NPB		
	$\hat{\beta }$	95% CI	PIP	$\hat{\beta }$	95% CI	PIP
C	0.00	(0.00, 0.04)	0.07	0.00	(0.00, 0.03)	0.07
MeBr	0.00	(-0.02, 0.00)	0.06	0.00	(-0.01, 0.00)	0.06
OP	0.02	(0.00, 0.12)	0.21	0.01	(0.00, 0.11)	0.16
O_3_	0.00	(-0.08, 0.02)	0.11	-0.01	(-0.12, 0.01)	0.11
NO_2_	-0.08	(-0.35, 0.00)	0.47	-0.12	(-0.36, 0.00)	0.60
PM_2.5_	0.00	(-0.08, 0.06)	0.13	0.00	(-0.09, 0.05)	0.12
PM_10_	0.02	(0.00, 0.21)	0.21	0.02	(-0.01, 0.20)	0.19

**Fig 1:**
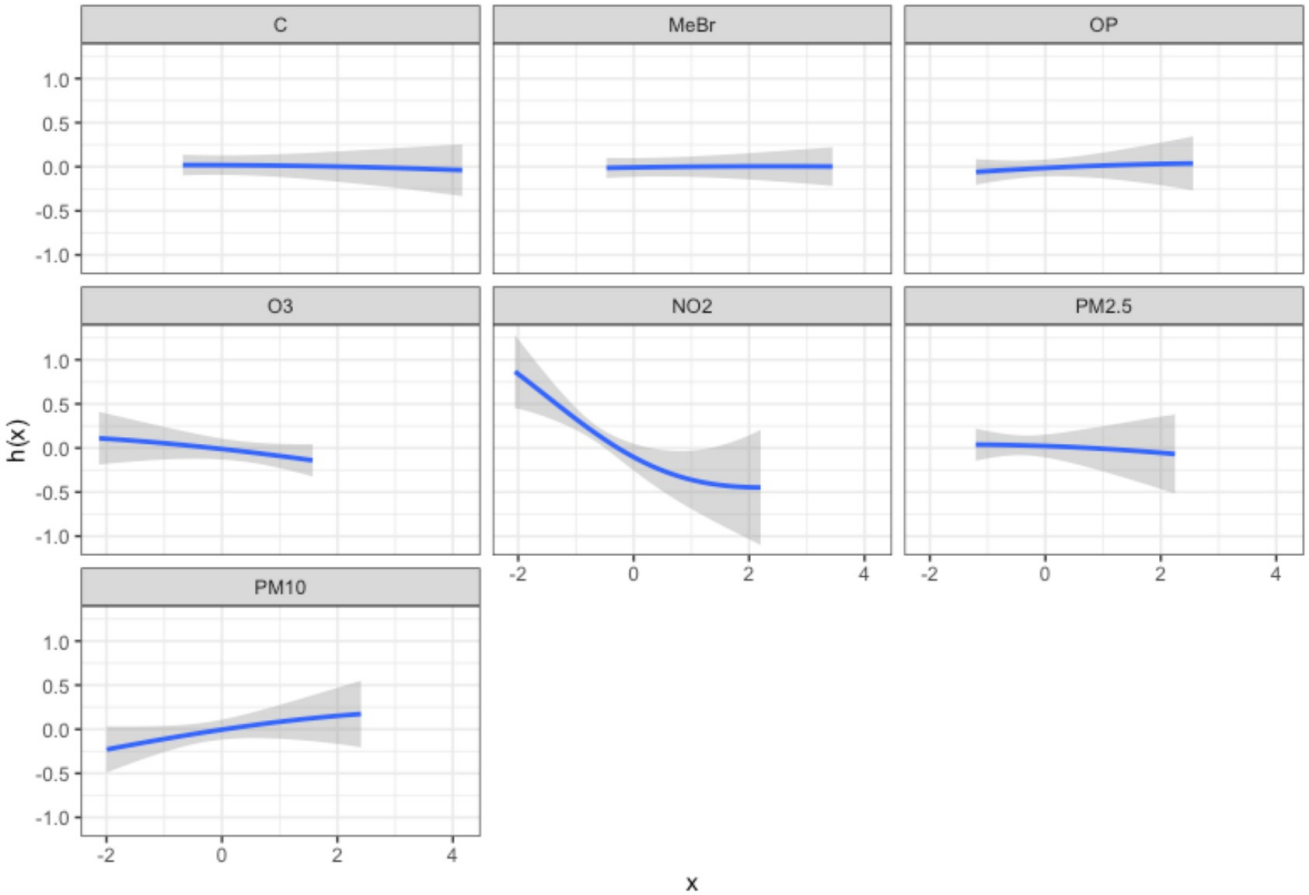
Results from analysis of FACES data set using BKMR. Figure shows the univariate relationship between each exposure and FEV_1_, holding all other exposures at their median value.

In BKMR, NO_2_ was selected as an important mixture component with a PIP of 0.96 (S11 Table in S1 File). No other exposures had PIPs above 0.5. Results were similar using the HVS formulation (S12 Table in S1 File). NO_2_ had a negative and nonlinear association with FEV_1_ (Fig 1). To identify interactions, we plot the posterior distribution of the exposure-response function for each pair of exposures, holding all other exposures constant at their median values, and visually inspect changes in the response as both exposures change. In doing so we found no notable interactions among exposures (S1 Fig in S1 File).

**Fig 2:**
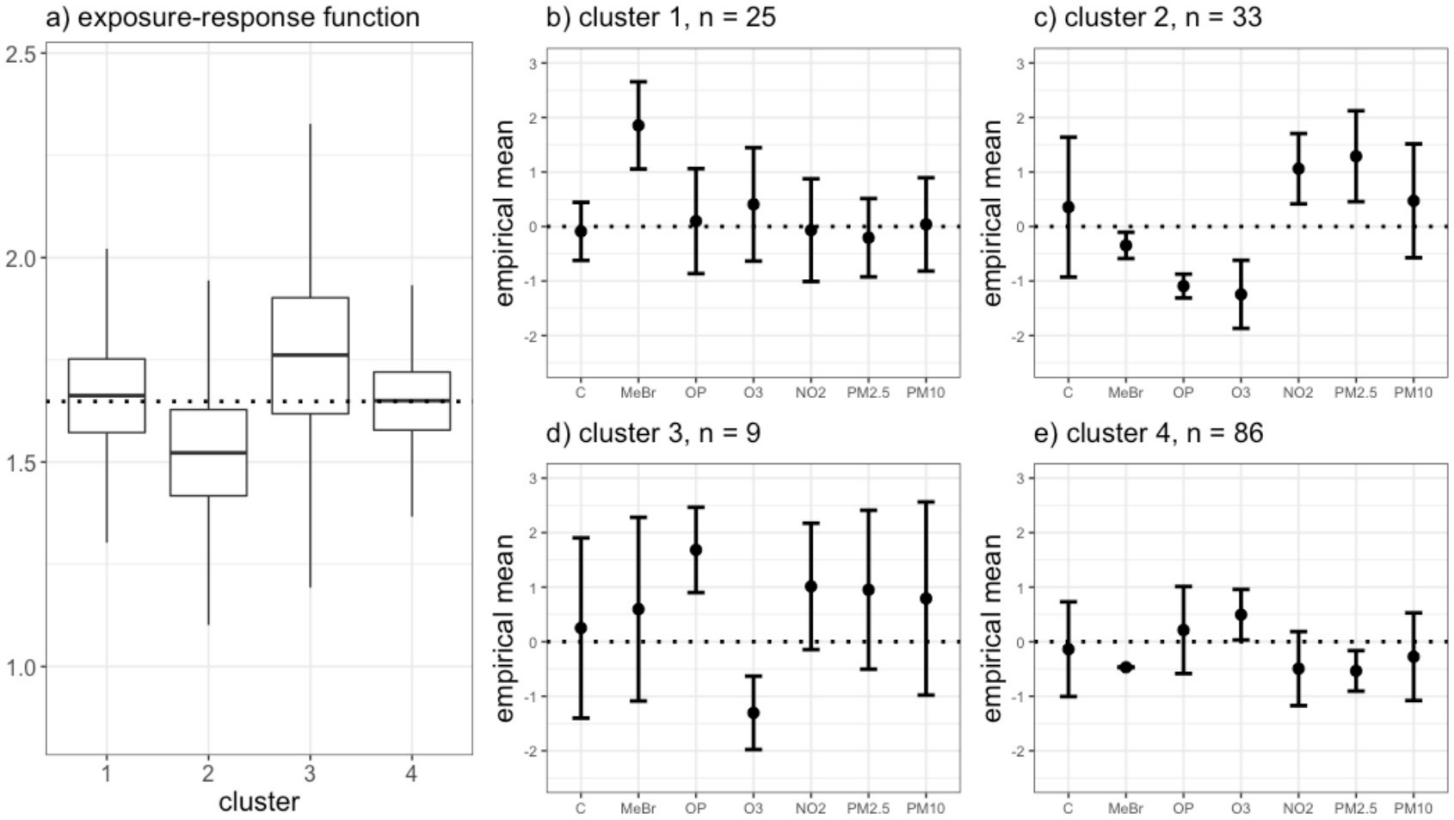
Results from analysis of FACES data set using UPR. Panel (a) shows the distribution of the model averaged estimated exposure-response function (*θ*_*c*_) for each cluster identified in the best clustering by UPR. The dotted line represents the overall mean estimated exposure-response function across all clusters. Panels (b-e) show the empirical exposure means of the individuals assigned to each cluster in the best clustering, with 1 standard deviation error bars. The dotted lines are drawn at 0, the mean of the standardized exposure data.

As clustering algorithms, UPR and SPR reveal a different kind of story. UPR revealed four clusters as the best partitioning of the data. Each cluster had similar estimated health effects (Fig 2a); hence, despite partitioning the exposure space there was no meaningful association between the exposure profiles and the health outcome. Fig 2b–2e shows the empirical exposure means for individuals assigned to each cluster. The first cluster of n = 25 individuals was distinguished by higher than average exposure to MeBr. Cluster 2 (n = 33) had low exposure to OP and O_3_ and high exposure to NO_2_ and PM_2.5_ relative to the average. The third cluster (n = 9) was characterized by relatively high exposure to OP and low exposure to O_3_. Individuals in cluster 4 (n = 86) had nearly average exposure to most pollutants except MeBR, which was notably below average; in addition, O_3_ exposure was slightly above and PM_2.5_ exposure was slightly below average. UPR selected OP (PIP = 0.57), O_3_ (PIP = 0.54), NO_2_ (PIP = 0.61), and PM_2.5_ (PIP = 0.56) as important mixture components (S13 Table in S1 File).

**Fig 3:**
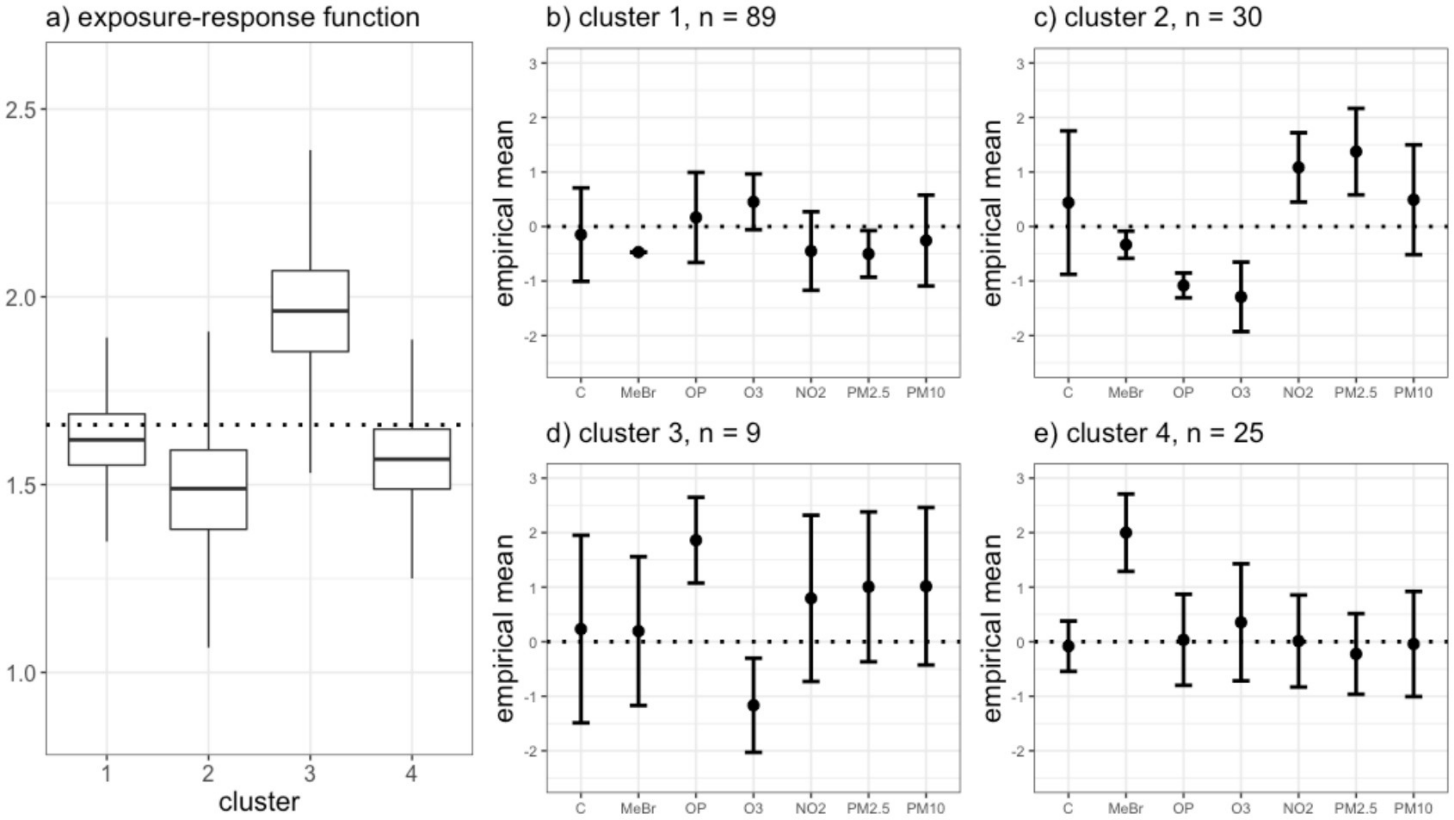
Results from analysis of FACES data set using SPR. Panel (a) shows the distribution of the model averaged estimated exposure-response function (*θ*_*c*_) for each cluster identified in the best clustering by SPR. The dotted line represents the overall mean estimated exposure-response function across all clusters. Panels (b-e) show the empirical exposure means of the individuals assigned to each cluster in the best clustering, with 1 standard deviation error bars. The dotted lines are drawn at 0, the mean of the standardized exposure data.

SPR also revealed four clusters as the best partitioning of the data. The estimated exposure-response function for cluster 3, the smallest cluster (*n* = 9), had a 0.97 posterior probability of being greater than the overall mean estimated exposure-response function (Fig 3a). The cluster sample sizes and associated empirical exposure means were very similar to those in UPR (Fig 3b–3e), with the labels switched for clusters 1 and 4. In both UPR and SPR, cluster 3 was the smallest cluster and had an estimated mean health effect higher than average, but there was more uncertainty around the health effect in UPR likely due to the two-stage approach for estimation. SPR selected five important mixture components: MeBr (PIP = 0.71), OP (PIP = 0.51), O_3_ (PIP = 0.75), NO_2_ (PIP = 0.67), and PM_2.5_ (PIP = 0.63) (S13 Table in S1 File). We found the clustering and PIPs in UPR and SPR to be sensitive to prior choice particularly for the cluster-specific precision matrix and error precision.

## Discussion

Interest is rapidly growing in estimating the association between exposure to mixtures of environmental chemicals and health outcomes. As a result, new statistical approaches have been developed for studying health outcomes associated with exposure to mixtures. The purpose of this paper was to evaluate and compare recently developed methods for mixtures and determine which research questions they answer well and in which scenarios. We limited our study to contemporary Bayesian methods since they are under-studied, under-utilized, and may have the ability to answer multiple research questions. Our results highlight the advantages of the flexible modeling and Bayesian framework of BKMR and NPB in estimating the exposure-response function precisely and identifying mixture components most strongly associated with the health outcome.

Overall, BKMR was a top-performing method. In each of the scenarios, BKMR estimated the exposure-response function with coverage closest to the nominal level (0.95) and lowest RMSE. Despite being a more flexible approach based on Gaussian processes, BKMR had lower RMSE in the linear scenario than NPBr, LM, and LM-int, all of which assume linearity. This is likely because NPBr and LM do not account for interactions and LM-int can result in inflated standard errors in the presence of correlated data. BKMR identified active mixture components with the greatest frequency, but also included inactive components more often than other methods. Although we did not evaluate variable selection rates for interactions in BKMR in our simulation, BKMR can identify linear or nonlinear interactions among exposures through visualization or summarizing the posterior distribution of the exposure-response function. A drawback to BKMR is that results are not as easily interpreted as in NPB or the linear models, though there are currently efforts to enhance interpretation and a suite of visualization approaches that aid in interpretation. BKMR is an appealing choice for mixtures because it makes minimal assumptions on the shape of the exposure-response function and includes a sophisticated variable selection algorithm for identifying important mixture components.

NPB was top-performing in the linear scenario regarding estimating the exposure-response function, identifying both active and inactive mixture components, and identifying interactions. NPB performed well even when the exposure-response function was mildly nonlinear, but lacks the flexibility of BKMR for the fixed profiles scenario, which is highly nonlinear. The AME method NPBr poorly estimated the exposure-response function in the linear scenario, likely from not accounting for interactions. An advantage of NPB is its ease of interpretation, which is similar to interpreting a linear regression model. NPB estimates PIPs and effect sizes for all main effect and interaction terms, providing precise information regarding the contribution of each exposure to the mixture and its effect on the health outcome.

The profile regression methods, UPR and SPR, poorly addressed the research questions of interest in all three scenarios. Two explanations for this include lack of a clustering structure in the exposure data and a weak signal, both of which inhibit these methods from accurately estimating the multipollutant exposure-response function. Further, UPR and SPR do not have the ability to identify or estimate interactions or tease out individual effects of the pollutants within a mixture. These methods may not be appropriate for studies in which the primary objectives are to estimate the multipollutant exposure-response function and identify driving mixture components. As clustering methods, UPR and SPR are likely to perform better on data that has a strong grouping structure. Since we used a single data set in our simulation study, the results of our simulation should not be interpreted as representative of performance on all data structures. A particular advantage of UPR and SPR is that the number of clusters need not be pre-specified.

The linear model with interactions, LM-int, had coverage above 0.91 in all three scenarios, but had higher RMSE and lower TSR than BKMR and NPB. LM-int and NPB are both EMM methods, and NPB outperformed LM-int in the linear EMM scenario. LM and LM-int have the advantage of being easy to implement and interpret, but these methods estimated the exposure-response function with more uncertainty than the top-performing methods and generally lacked the ability to select truly active mixture components, likely due to high correlation among exposures.

In our application to the FACES data set, four methods (LM, LM-int, NPB, and BKMR) identified NO_2_ as an important mixture component negatively associated with the health outcome. In addition, LM and LM-int estimated PM_10_ to have a positive association with FEV_1_, and PM_10_ was positively correlated with NO_2_. Further, the magnitude of the effect estimate for NO_2_ in LM and LM-int was several times larger than that estimated in NPB, and the confidence intervals were also larger, reflecting more uncertainty. UPR and SPR also identified NO_2_ as an important mixture component, but we cannot determine the sign of effect using these methods. Instead, UPR and SPR have the ability to estimate how the overall mixture is associated with the health outcome. UPR revealed four clusters with similar estimated health effects; hence, patterns in the exposure data were not associated with FEV_1_. In SPR, the smallest cluster was associated with higher average FEV_1_ than the other clusters, suggesting an association between a relatively rare mixture of exposures and the health outcome. Alternatively, this small cluster may reflect a strong influence from the health outcome in the clustering using a supervised learner. Meanwhile, BKMR was able to describe a nonlinear association between NO_2_ and FEV_1_.

Using missing indicators may have introduced some bias in the effect estimates. Additionally, all Bayesian methods are sensitive to prior specification and results may vary with more or less informative priors. PIPs are particularly sensitive to prior specification in all methods, so changing prior hyperparameters may lead to changes in TSR and FSR. We implemented all models using the default priors as specified by the authors to obtain an objective comparison of these methods.

Along with the primary research question, the best performing method is likely to depend on the exposure data. We used observed exposure data so our results are highly relevant to realistic settings. Our simulation results can be generalized to small data sets with a limited number of localized exposures, which is a frequent scenario in epidemiological studies.

In analyses of environmental mixtures and human health, model choice depends on the assumed exposure-response relationship and the primary questions of interest. NPB and BKMR are recently proposed methods that outperformed traditional regression models and offer promising tools for mixtures analyses. We recommend NPB when the exposure-response function is assumed to be approximately linear and a primary goal is accurately identifying which are the active and inactive components of the mixture. NPB is also highly interpretable and explicitly tests for interactions. We recommend BKMR if the exposure-response function is assumed to take on a complex form and the primary goal is estimating the form of the exposure-response function while at the same time identifying important mixture components. Our results suggest that UPR and SPR do not reliably answer our specified research questions, but may be applicable for different research questions such as pattern recognition. We further encourage users to take advantage of our R package mmpack [44] to replicate the simulation and determine how each method performs on their own data. Results will likely be different on different data sets. In particular, the profile regression methods may perform better on data that exhibits a stronger clustering structure in the fixed profiles scenario. We include the clustering statistics as part of the summary of the fixed profile scenario output so users can see how much grouping structure is in their own data. Replicating the simulation on their own data will enable users to choose the best method for their data and specific research question.

## Supporting information

S1 File (PDF)
