# Supplementary material for: Model choice for estimating the association between exposure to chemical mixtures and health outcomes: A simulation study
Source: PLoS One. 2021 Mar 25;16(3):e0249236. doi: 10.1371/journal.pone.0249236 (PMC7993848; doi:10.1371/journal.pone.0249236)
Supplement: S1 File — (PDF) [file pone.0249236.s001.pdf]

Supporting information

S1 Table. Descriptive statistics of FACES data. Demographic characteristics of the sample of FACES data used in simulation studies and data analysis.

	n = 153
Age (years), mean (SD)	9 (1.8)
Male gender, %	60.1
Height (inches), mean (SD)	52.3 (4.8)
Ethnicity, %	
Non-Hispanic Black	13.7
Non-Hispanic White	46.4
Hispanic	39.9
BMI (kg/m^2), mean (SD)	18.5 (4.6)
Mother $<$ 12th grade education, %	60.1
Insured, %	94.8
Atopy, %	78.4
Father/Mother smokes currently, %	5.2
Proximity to Freeway ($<$ 1 block away), %	47.7
Severity (GINA $\geq$ 3), %	17.6
Household income $>$ 30K/year, %	52.3
FEV_1 (L), mean (SD)	1.7 (0.4)

S2 Table. Grouping structure in fixed profiles scenario. Table shows summary statistics for the Calinski-Harabasz index, silhouette statistic, and number of clusters to maximize the gap width (Gap clusters) for 200 simulated data sets used in the simulation study.

	Min	1st quartile	Median	Mean	3rd quartile	Max
Calinski-Harabasz	6.0647	18.1074	22.5437	21.3772	25.9485	35.0493
Silhouette	0.0015	0.1099	0.1463	0.1515	0.1878	0.2900
Gap clusters	2	4	6	6.07	9	10

S3 Table. Summary of method performance in the linear scenario. Results from simulation study across 200 simulated data sets in scenario h_1: linear. Reported values are means (standard errors) across all data sets for: root mean squared error (RMSE) and coverage (Cvg) for the exposure-response function, true selection rate for main effects (TSR), false selection rate for main effects (FSR), true selection rate for interactions (TSR_{int}), and false selection rate for interactions (FSR_{int}).

	NPBr	NPB	UPR	SPR
RMSE	1.02 (0.02)	0.54 (0.01)	2.01 (0.04)	1.59 (0.04)
Cvg	0.73 (0.01)	0.95 (0.01)	0.56 (0.01)	0.54 (0.01)
TSR	0.85 (0.01)	0.92 (0.01)	0.25 (0.02)	0.63 (0.02)
FSR	0.35 (0.02)	0.10 (0.01)	0.26 (0.02)	0.53 (0.02)
TSR_{int}	–	0.59 (0.02)	–	–
FSR_{int}	–	0.02 (0.00)	–	–

	BKMR	LM	LM-int
RMSE	0.55 (0.01)	1.01 (0.02)	0.73 (0.01)
Cvg	0.96 (0.01)	0.73 (0.01)	0.95 (0.01)
TSR	1.00 (0.00)	0.84 (0.01)	0.68 (0.01)
FSR	0.39 (0.02)	0.29 (0.02)	0.04 (0.01)
TSR_{int}	–	–	0.32 (0.02)
FSR_{int}	–	–	0.04 (0.00)

S4 Table. Summary of method performance in the nonlinear scenario. Results from simulation study across 200 simulated data sets in scenario h_2: nonlinear. Reported values are means (standard errors) across all data sets for: root mean squared error (RMSE) and coverage (Cvg) for the exposure-response function, true selection rate for main effects (TSR), false selection rate for main effects (FSR), true selection rate for interactions (TSR_{int}), and false selection rate for interactions (FSR_{int}).

	NPBr	NPB	UPR	SPR
RMSE	0.77 (0.01)	0.69 (0.01)	1.42 (0.03)	1.27 (0.03)
Cvg	0.80 (0.01)	0.86 (0.01)	0.56 (0.01)	0.58 (0.01)
TSR	0.79 (0.02)	0.78 (0.02)	0.27 (0.02)	0.68 (0.02)
FSR	0.22 (0.02)	0.16 (0.02)	0.24 (0.02)	0.58 (0.02)
TSR_{int}	–	0.25 (0.03)	–	–
FSR_{int}	–	0.01 (0.00)	–	–

	BKMR	LM	LM-int
RMSE	0.59 (0.01)	0.78 (0.01)	0.89 (0.02)
Cvg	0.92 (0.01)	0.81 (0.01)	0.91 (0.01)
TSR	0.96 (0.01)	0.78 (0.02)	0.54 (0.02)
FSR	0.48 (0.02)	0.17 (0.01)	0.08 (0.01)
TSR_{int}	–	–	0.20 (0.03)
FSR_{int}	–	–	0.07 (0.01)

S5 Table. Summary of method performance in the fixed profiles scenario. Results from simulation study across 200 simulated data sets in scenario h_3: fixed profiles. Reported values are means (standard errors) across all data sets for: root mean squared error (RMSE) and coverage (Cvg) for the exposure-response function, true selection rate for main effects (TSR), false selection rate for main effects (FSR), true selection rate for interactions (TSR_{int}), and false selection rate for interactions (FSR_{int}).

	NPBr	NPB	UPR	SPR
RMSE	1.11 (0.02)	1.02 (0.02)	1.41 (0.02)	1.38 (0.02)
Cvg	0.66 (0.01)	0.75 (0.01)	0.55 (0.01)	0.54 (0.01)
TSR	0.66 (0.02)	0.68 (0.02)	0.27 (0.03)	0.68 (0.02)
FSR	0.11 (0.01)	0.13 (0.01)	0.25 (0.02)	0.59 (0.01)
TSR_{int}	–	0.06 (0.02)	–	–
FSR_{int}	–	0.02 (0.00)	–	–

	BKMR	LM	LM-int	
RMSE	0.69 (0.01)	1.13 (0.02)	0.99 (0.02)	
Cvg	0.91 (0.01)	0.70 (0.01)	0.91 (0.00)	
TSR	0.97 (0.01)	0.69 (0.02)	0.56 (0.02)	
FSR	0.64 (0.03)	0.14 (0.01)	0.14 (0.01)	
TSR_{int}	–	–	0.12 (0.02)	
FSR_{int}	–	–	0.11 (0.01)	

S6 Table. Summary of method performance in the null scenario. Results from simulation study across 100 simulated data sets in the null scenario. Reported values are means (standard errors) across all data sets for: root mean squared error (RMSE) and coverage (Cvg) for the exposure-response function, false selection rate for main effects (FSR), and false selection rate for interactions (FSR_{int}). True selection rates were not reported since there were no active mixture components in the exposure-response function.

	NPBr	NPB	UPR	SPR
RMSE	0.23 (0.02)	0.24 (0.02)	0.28 (0.02)	0.56 (0.06)
Cvg	0.98 (0.02)	0.98 (0.02)	0.98 (0.01)	0.74 (0.05)
FSR	0.00 (0.00)	0.00 (0.00)	0.28 (0.03)	0.74 (0.02)
FSR_{int}	–	0.00 (0.00)	–	–

	BKMR	LM	LM-int	
RMSE	0.25 (0.02)	0.44 (0.02)	0.77 (0.03)	
Cvg	0.97 (0.01)	0.96 (0.01)	0.95 (0.01)	
FSR	0.30 (0.04)	0.03 (0.01)	0.08 (0.01)	
FSR_{int}	–	–	0.07 (0.01)	

S7 Table. Summary of method performance in the complex mixture scenario. Results from simulation study across 100 simulated data sets in the complex mixture scenario. Reported values are means (standard errors) across all data sets for: root mean squared error (RMSE) and coverage (Cvg) for the exposure-response function, true selection rate for main effects (TSR), false selection rate for main effects (FSR), true selection rate for interactions (TSR_{int}), and false selection rate for interactions (FSR_{int}).

	NPBr	NPB	UPR	SPR
RMSE	1.00 (0.03)	0.69 (0.03)	3.22 (0.12)	2.97 (0.12)
Cvg	0.77 (0.02)	0.91 (0.01)	0.46 (0.01)	0.32 (0.02)
TSR	0.62 (0.02)	0.58 (0.02)	0.00 (0.00)	0.29 (0.04)
FSR	0.19 (0.03)	0.10 (0.02)	0.00 (0.00)	0.31 (0.05)
TSR_{int}	–	0.39 (0.03)	–	–
FSR_{int}	–	0.01 (0.00)	–	–

	BKMR	LM	LM-int
RMSE	0.86 (0.05)	1.00 (0.03)	1.68 (0.06)
Cvg	0.90 (0.01)	0.80 (0.02)	0.96 (0.01)
TSR	0.65 (0.02)	0.56 (0.01)	0.23 (0.01)
FSR	0.28 (0.04)	0.08 (0.01)	0.04 (0.01)
TSR_int	–	–	0.07 (0.02)
FSR_int	–	–	0.04 (0.01)

S8 Table. Summary of method performance in large sample size ($n = 1000$) simulation study. Results from the large sample size simulation study across 100 simulated data sets in all three exposure-response scenarios. Reported values are means across all data sets for: root mean squared error (RMSE) and coverage (Cvg) for the exposure-response function, true selection rate for main effects (TSR), false selection rate for main effects (FSR), true selection rate for interactions (TSR_{int}), and false selection rate for interactions (FSR_{int}). Results for top-performing methods are listed in **bold**.

Method	RMSE	Cvg	TSR	FSR	TSR_{int}	FSR_{int}
$h_1(\mathbf{x})$: linear with multiplicative interactions						
NPBr	0.91	0.37	0.96	0.69	–	–
NPB	**0.14**	**0.96**	**1.00**	**0.01**	**1.00**	**0.00**
UPR	1.53	0.28	**1.00**	1.00	–	–
SPR	1.40	0.28	**1.00**	1.00	–	–
BKMR	0.23	**0.96**	**1.00**	0.04	–	–
LM	0.90	0.37	0.96	0.68	–	–
LM-int	0.30	**0.95**	**0.99**	0.04	0.92	0.06
$h_2(\mathbf{x})$: nonlinear with multiplicative interactions						
NPBr	0.65	0.45	0.96	0.44	–	–
NPB	0.48	0.67	0.96	**0.24**	**0.74**	**0.20**
UPR	1.08	0.32	**0.99**	1.00	–	–
SPR	1.10	0.33	**1.00**	1.00	–	–
BKMR	**0.29**	**0.92**	**1.00**	**0.25**	–	–
LM	0.65	0.46	0.95	0.49	–	–
LM-int	0.58	0.71	0.86	0.27	0.62	0.25
$h_3(\mathbf{x})$: constant function of fixed profiles						
NPBr	1.08	0.33	0.87	**0.50**	–	–
NPB	0.75	0.57	0.93	**0.51**	0.54	**0.39**
UPR	1.15	0.35	**0.99**	1.00	–	–
SPR	1.17	0.35	**0.99**	1.00	–	–
BKMR	**0.43**	**0.87**	**0.99**	0.67	–	–
LM	1.09	0.33	0.89	0.54	–	–
LM-int	0.77	0.63	0.83	0.53	**0.58**	0.44

S9 Table. Additional results from analysis of FACES data set using LM-int. Table shows main effect and interaction regression coefficient estimates ($\hat{\beta}$), 95% confidence intervals (CI), and *p*-values. The regression coefficient $\hat{\beta}$ is the expected change in FEV_1 for a 1 standard deviation increase in the square root transformed exposures.

	$\hat{\beta}$	95% CI	*p*-value
**Main Effects**			
C	0.05	(-0.08 , 0.19)	0.44
MeBr	0.17	(0.05 , 0.29)	0.01
OP	0.02	(-0.17 , 0.22)	0.80
O_3	-0.13	(-0.32 , 0.06)	0.17
NO_2	-0.68	(-1.10 , -0.25)	0.00
$PM_{2.5}$	-0.11	(-0.48 , 0.26)	0.55
PM_{10}	0.50	(0.08 , 0.93)	0.02
**Interactions**			
C:MeBr	-0.04	(-0.14 , 0.07)	0.51
C:OP	0.15	(-0.18 , 0.47)	0.38
C:O_3	-0.01	(-0.18 , 0.16)	0.91
C:NO_2	-0.06	(-0.35 , 0.23)	0.67
C:$PM_{2.5}$	0.28	(0.01 , 0.54)	0.04
C:PM_{10}	-0.08	(-0.31 , 0.14)	0.48
MeBr:OP	0.01	(-0.26 , 0.28)	0.93
MeBr:O_3	-0.03	(-0.20 , 0.15)	0.77
MeBr:NO_2	-0.11	(-0.43 , 0.21)	0.50
MeBr:$PM_{2.5}$	0.18	(-0.06 , 0.42)	0.14
MeBr:PM_{10}	0.08	(-0.11 , 0.28)	0.41
OP:O_3	-0.04	(-0.20 , 0.12)	0.63
OP:NO_2	-0.10	(-0.33 , 0.12)	0.37
OP:$PM_{2.5}$	-0.23	(-0.58 , 0.12)	0.19
OP:PM_{10}	0.31	(-0.01 , 0.62)	0.05
O_3:NO_2	-0.12	(-0.54 , 0.29)	0.56
O_3:$PM_{2.5}$	0.04	(-0.23 , 0.30)	0.78
O_3:PM_{10}	-0.02	(-0.31 , 0.27)	0.88
NO_2:$PM_{2.5}$	-0.27	(-0.70 , 0.16)	0.21
NO_2:PM_{10}	0.33	(-0.05 , 0.72)	0.09
$PM_{2.5}$:PM_{10}	0.01	(-0.38 , 0.40)	0.95

S10 Table. Additional results from analysis of FACES data set using NPB. Table shows main effect and interaction regression coefficient estimates ($\hat{\beta}$), 95% credible intervals, and posterior inclusion probabilities (PIP). The regression coefficient $\hat{\beta}$ is the expected change in FEV_1 for a 1 standard deviation increase in the square root transformed exposures.

	$\hat{\beta}$	95% CI	PIP
**Main Effects**			
C	0.00	(0.00 , 0.03)	0.07
MeBr	0.00	(-0.01 , 0.00)	0.06
OP	0.01	(0.00 , 0.11)	0.16
O_3	-0.01	(-0.12 , 0.01)	0.11
NO_2	-0.12	(-0.36 , 0.00)	0.60
$PM_{2.5}$	0.00	(-0.09 , 0.05)	0.12
PM_{10}	0.02	(-0.01 , 0.2)	0.19
**Interactions**			
C:MeBr	0.00	(0.00 , 0.00)	0.02
C:OP	0.00	(0.00 , 0.00)	0.02
C:O_3	0.00	(0.00 , 0.00)	0.01
C:NO_2	0.00	(0.00 , 0.00)	0.01
C:$PM_{2.5}$	0.00	(0.00 , 0.00)	0.01
C:PM_{10}	0.00	(0.00 , 0.00)	0.01
MeBr:OP	0.00	(0.00 , 0.00)	0.01
MeBr:O_3	0.00	(0.00 , 0.00)	0.01
MeBr:NO_2	0.00	(0.00 , 0.00)	0.01
MeBr:$PM_{2.5}$	0.00	(0.00 , 0.00)	0.02
MeBr:PM_{10}	0.00	(0.00 , 0.00)	0.02
OP:O_3	0.00	(0.00 , 0.00)	0.02
OP:NO_2	0.00	(0.00 , 0.00)	0.01
OP:$PM_{2.5}$	0.00	(0.00 , 0.00)	0.01
OP:PM_{10}	0.00	(0.00 , 0.00)	0.01
O_3:NO_2	0.00	(0.00 , 0.00)	0.01
O_3:$PM_{2.5}$	0.00	(0.00 , 0.00)	0.01
O_3:PM_{10}	-0.01	(-0.09 , 0.00)	0.06
NO_2:$PM_{2.5}$	0.01	(0.00 , 0.16)	0.11
NO_2:PM_{10}	0.01	(0.00 , 0.13)	0.12
$PM_{2.5}$:PM_{10}	0.00	(0.00 , 0.06)	0.05

S11 Table. Variable selection results from FACES data analysis using BKMR with component-wise variable selection. Table shows posterior inclusion probabilities (PIP) for each exposure.

Exposure	PIP
C	0.08
MeBr	0.06
OP	0.10
O_3	0.16
NO_2	0.96
$PM_{2.5}$	0.20
PM_{10}	0.34

S12 Table. Variable selection results from FACES data analysis using BKMR with hierarchical variable selection. Table shows posterior inclusion probabilities for each group (Group PIP) as well as conditional posterior inclusion probabilities for each exposure given the group to which it belongs is included (Conditional PIP). Component-wise PIPs are calculated from the group and conditional PIPs by multiplying the group PIP by the conditional PIP for each exposure.

Exposure	Group PIP	Conditional PIP	Component-wise PIP
C	0.20	0.18	0.03
MeBr	0.20	0.13	0.03
OP	0.20	0.70	0.14
O_3	0.98	0.00	0.00
NO_2	0.98	0.98	0.96
$PM_{2.5}$	0.98	0.01	0.01
PM_{10}	0.98	0.00	0.00

S1 Fig. Estimated bivariate exposure-response function from FACES data analysis using BKMR. Each grid panel is an image plot of the predicted exposure-response function $\hat{h}$ for varying levels of two exposures, while holding all other exposures at their median value. Evidence of an interaction would be reflected by changes in the predicted exposure-response function with changes in the levels of both exposure 1 and exposure 2. Figure shows no notable evidence of interactions, but does depict the main effect of NO_2.

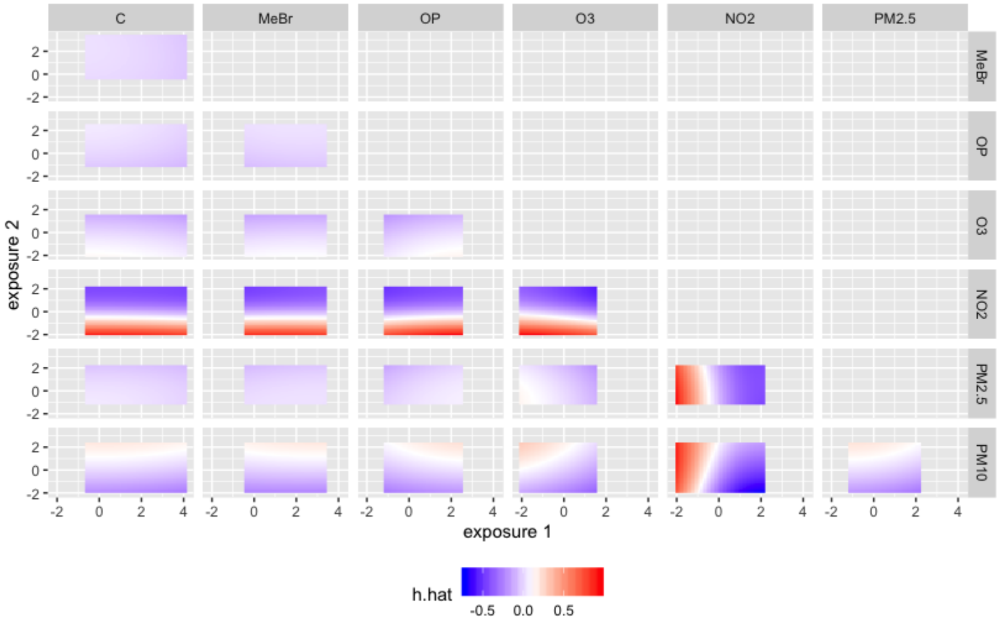

S13 Table. Variable selection results from FACES data analysis using UPR and SPR.
Table shows posterior inclusion probabilities (PIP) for each exposure in each method.

	PIP	
Exposure	UPR	SPR
C	0.03	0.02
MeBr	0.21	0.71
OP	0.57	0.51
O_3	0.54	0.75
NO_2	0.61	0.67
$PM_{2.5}$	0.56	0.63
PM_{10}	0.24	0.03

S1 Appendix: Additional simulations

Simulation design

We conduct three additional simulation studies to assess robustness of our results. For all of the additional simulations, we report results from 100 simulated data sets.

First, we include a null scenario, $h_4(\mathbf{x})$, where none of the exposures are associated with the response. That is,

$$h_4(\mathbf{x}) = 0. \tag{1}$$

This scenario uses the same exposure data, covariates, and residual variance described in scenarios 1-3 in the main text.

Second, we include a complex mixtures scenario, $h_5(\mathbf{x})$, where we simulate data for seven additional pollutants to have a total of 14 mixture components. For each data set, the first seven pollutants are the exposures from the FACES data set as described in scenarios 1-3 in the main text. The exposure values for the seven additional pollutants are simulated as random linear combinations of the FACES exposure data using N(0,1) weights plus N(0,1) noise. All exposures are then scaled to have mean 0 and variance 1. We simulate the response as a linear function of 10 main effects and two pairwise interactions. Specifically,

$$\begin{aligned} h_5(\mathbf{x}) \quad = \quad & x_1 - x_2 + x_3 - x_4 + 1.4x_5 + 1.5x_6 + 1.2x_7 - \\ & 1.4x_8 - 1.5x_9 - 1.2x_{10} + 0.7x_1x_2 - 0.5x_3x_4. \end{aligned} \tag{2}$$

The ten active mixture components $x_1, \ldots, x_{10}$ are randomly selected for each data set. All 14 pollutants are included in the models as predictors. All other details of the data generating mechanism are the same as previously described for the other scenarios.

Third, we replicate the simulation scenarios 1-3 in the main text but use a larger sample size. We repeatedly sample from the FACES exposure and covariate data to create a sample of size $n = 1000$ for each data set. All other details are described in the main text.

Simulation results

The methods performed more similarly to each other in the null scenario compared to the other scenarios (S6 Table). NPBr, NPB, and BKMR had the lowest RMSE for the exposure-response function and LM-int had the highest RMSE. All methods except SPR achieved the nominal coverage level. FSR was lowest for NPBr and NPB, meaning these methods were the best at not selecting any mixture components into the model when none are associated with the response. FSR was highest for SPR.

Results from the complex mixture scenario are shown in S7 Table. Here NPB estimated the exposure-response function with lowest RMSE and near-optimal coverage. BKMR had the next lowest RMSE. LM-int achieved the nominal coverage, but with substantially higher RMSE. NPBr and BKMR had highest TSR, followed by NPB and LM. NPB, UPR, LM, and LM-int all had mean FSR at or below 0.10. NPB outperformed LM-int in variable selection rates for interactions. Overall, NPB and BKMR were the top-performing methods in simultaneously estimating the exposure-response function and identifying active mixture components in this complex mixture scenario.

For the larger sample size simulation, our results remain generally the same as in our original simulation study (S8 Table). NPB performed best in the linear scenario, followed by BKMR and LM-int. BKMR performed best in the nonlinear and fixed profiles scenarios. TSR improved for all methods in all scenarios. With the increased sample size, UPR and SPR often selected all of the mixture components into the model, as evidenced by both high TSR and FSR.

S2 Appendix: Additional model details

Nonparametric Bayes shrinkage

Nonparametric Bayes shrinkage was originally introduced as a method in the logistic regression setting [1], and we adapted it for use in a linear regression setting for analysis of a continuous health outcome. The original model includes main effects and all pairwise interactions (NPB). We also implemented a reduced model with main effects only (NPBr).

To introduce the model, denote a continuous response y_i, exposures $x_{ij}, j = 1, \ldots, p$, and covariates, $w_{il}, l = 1, \ldots q$. The response is modeled as

$$y_i = \gamma_0 + \sum_{j=1}^{p} x_{ij}\beta_j + \sum_{j=1}^{p-1}\sum_{k=j+1}^{p} x_{ij}x_{ik}\zeta_{jk} + \sum_{l=1}^{q} w_{il}\gamma_l + \varepsilon_i,$$

where γ_0 is an overall intercept and $\varepsilon_i \overset{iid}{\sim} N(0, \sigma^2)$ for $i = 1, \ldots, n$. Equivalently, the model can be expressed as

$$y_i|\beta, \gamma, \zeta, \sigma^2 \sim \mathrm{N}\left(\gamma_0 + \mathbf{x}_i^T\beta + \mathbf{z}_i^T\zeta + \mathbf{w}_i^T\gamma, \sigma^2\right),$$

where $\mathbf{x}_i$ denotes the vector of exposures, $\mathbf{w}_i$ denotes the vector of covariates, and $\mathbf{z}_i$ denotes the vector of the pairwise multiplicative interactions between elements in $\mathbf{x}_i$.

A Dirichlet process (DP) prior is placed on the main effect regression coefficients β. The base distribution is a mixture of a normal distribution and a point mass at 0 to induce sparsity in the model and perform variable selection. We assign a normal-inverse gamma prior to the mean and variance of the normal base measure. A separate, but similarly constructed, DP prior is placed on the pairwise multiplicative interaction coefficients ζ, where ζ_{jk} denotes the regression coefficient for the interaction between exposures j and k.

The models for main effects and interactions are described in the main text. The intercept, regression coefficients for covariates, and error variance are modeled

$$\begin{aligned} \gamma_0 &\sim N(\mu_0, \kappa_0^2) \\ \gamma|\mu_\gamma, \kappa^2 &\sim \mathrm{N}(\mu_\gamma, \kappa^2\mathbf{I}) \\ \sigma^{-2} &\sim \mathrm{Gamma}(\alpha_\sigma, \beta_\sigma). \end{aligned}$$

We set the following prior hyperparameters in our simulation study and data analysis:

$$\begin{array}{cccc} \mu_\gamma = \mathbf{0} & \kappa^{-2} = 1 & \mu_0 = 0 & \kappa_0^{-2} = 1 \\ \sigma_{\mu 1}^{-2} = 1 & \sigma_{\mu 2}^{-2} = 1 & \alpha_{\phi 1} = 1 & \beta_{\phi 1} = 1 \\ \alpha_{\phi 2} = 1 & \beta_{\phi 2} = 1 & \alpha_\sigma = 1 & \beta_\sigma = 1 \\ \alpha_{\pi 1} = 1 & \beta_{\pi 1} = 1 & \alpha_{\pi 2} = 9 & \beta_{\pi 2} = 1 \\ \alpha_{\alpha 1} = 2 & \beta_{\alpha 1} = 1 & \alpha_{\alpha 2} = 2 & \beta_{\alpha 2} = 1. \end{array}$$

Bayesian profile regression

Bayesian profile regression was originally introduced in the logistic regression setting [2, 3] and was later adapted to the linear regression context [4]. The original model is supervised profile regression, in which the response informs cluster assignment. We included an unsupervised version in which the response does not inform cluster assignment but is simultaneously estimated via Bayesian linear regression. The only difference between the supervised and unsupervised versions exists in the computation of the posterior distribution.

We fit supervised profile regression using the R package PReMiuM [5] and implement the truncated stick-breaking approach to approximate an infinite mixture with a finite one. See

Liverani et al [5] for details on the profile regression model with continuous exposures and a continuous health outcome.

We fit the unsupervised profile regression model using the R package mmpack [6]. We follow the same methods for model fitting and post-processing as in supervised profile regression and incorporate only one change in the computation of the posterior distribution so that the clustering is independent of the health outcome.

Let $\mathbf{x}_i = (x_{i1}, \ldots, x_{ip})^T$ be a vector of continuous exposures for individual i. Then the likelihood of an individual exposure profile can be written

$$f(\mathbf{x}_i|\psi, \mu_1, \ldots, \mu_C, \Sigma_1, \ldots, \Sigma_C) = \sum_{c=1}^{C} \psi_c f(\mathbf{x}_i|\mu_c, \Sigma_c). \quad (3)$$

Conditional on cluster assignment, $z_i = c$, the likelihood of an individual exposure profile is

$$\mathbf{x}_i|z_i = c, \mu_c, \Sigma_c \sim \mathrm{N}(\mu_c, \Sigma_c).$$

The remainder of the profile assignment model is described in the main text. We simultaneously model the health outcome y_i as a function of the cluster-specific intercept θ_{z_i} and covariates $\mathbf{w}_i = (w_{i1}, \ldots w_{iq})^T$:

$$\begin{aligned}
y_i|z_i = c, \theta, \gamma, \sigma^2 &\sim \mathrm{N}(\theta_c + \mathbf{w}_i^T \gamma, \sigma^2) \\
\theta_c|\kappa_c^{-2} &\sim \mathrm{N}(0, \kappa_c^2) \\
\kappa_c^{-2} &\sim \mathrm{Gamma}(\alpha_\kappa, \beta_\kappa) \\
\gamma_l|\phi_l^{-2} &\sim \mathrm{N}(0, \phi_l^2) \qquad l = 1, \ldots q \\
\phi_l^{-2} &\sim \mathrm{Gamma}(\alpha_\phi, \beta_\phi) \\
\sigma^{-2} &\sim \mathrm{Gamma}(\alpha_\sigma, \beta_\sigma).
\end{aligned}$$

See Liverani et al [5] for details on the binary cluster variable selection algorithm implemented here. The variable selection model is

$$\begin{aligned}
\mu^*_{c,j} &= \pi_{c,j}\mu_{c,j} + (1 - \pi_{c,j})\bar{x}_j \\
\pi_{c,j}|\rho_j &\sim \mathrm{Bernoulli}(\rho_j) \\
\rho_j|\omega_j &\sim I(\omega_j = 0)\delta_0(\rho_j) + I(\omega_j = 1)\mathrm{Beta}(\alpha_\rho, \beta_\rho) \\
\omega_j &\sim \mathrm{Bernoulli}(0.5).
\end{aligned}$$

If variable selection is implemented, as was done in both the simulation and data analysis, then μ_c^* replaces μ_c in the likelihood in equation 3.

Following Molitor et al [3], we set the following prior hyperparameters for our simulation and data analysis:

$$\begin{array}{cccc}
\alpha_\alpha = 2 & \beta_\alpha = 1 & \alpha_\kappa = \frac{7}{2} & \beta_\kappa = \frac{43.75}{2} \\
\alpha_\phi = \frac{7}{2} & \beta_\phi = \frac{43.75}{2} & \alpha_\sigma = 2.5 & \beta_\sigma = 2.5 \\
\alpha_\rho = 0.5 & \beta_\rho = 0.5 & r = p & C = 20.
\end{array}$$

We also set ν_0 as the vector of empirical exposure means and Λ_0 as a diagonal matrix where each non-zero element is the square of the observed range for each exposure. Further, R is set to the empirical covariance matrix of the exposure data.

The Bayesian framework of this model allows the number and size of clusters to vary across iterations. When making inference, we must carefully consider the uncertainty regarding the clustering. To do so, we follow the steps defined in Molitor et al. [3] and first determine the most optimal clustering of the data using a least squares distance algorithm adopted from

Dahl [7]. We construct an $n \times n$ score matrix at each iteration with a 1 in the i, j location if individuals i and j belong to the same cluster and a 0 otherwise. We then calculate a probability matrix $\mathbf{S}$ by averaging the score matrices. The most optimal clustering minimizes the least squared distance to $\mathbf{S}$. Details regarding this algorithm can be found elsewhere [2].

The primary parameter of interest in this model is θ_c. We implement model averaging techniques account for uncertainty in the clustering when estimating θ_c. In a post-processing step, we calculate the model-averaged estimates of θ_c in the "best" clustering of the data. For iteration s and cluster c, the model averaged estimates are calculated as

$$\bar{\theta}_c^{(s)} = \frac{1}{n_c} \sum_{i:z_i^{\text{best}}=c} \theta_c^{(s)},$$

where n_c is the number of individuals assigned to cluster c in the best clustering of the data (z^{best}). We then calculate the posterior mean, variance, and credible intervals for $\bar{\theta}_c$ to summarize the model averaged posterior health effect estimates for the best clusters.

We found UPR and SPR to be highly sensitive to prior specification with regards to PIPs and clustering. The stick-breaking process depends on the ordering of cluster labels but the likelihood of the DP mixture model does not; thus, SPR can suffer from poor mixing, motivating the use of label switching moves. Convergence can still be problematic and is difficult to check. Liverani et al. provides some diagnostic tools in the package PReMiuM [5].

Bayesian kernel machine regression

See Bobb et al. [8] and Bobb [9] for details on implementing Bayesian kernel machine regression. We use all default parameters in our simulation and data analysis.

References

1. Herring AH. Nonparametric bayes shrinkage for assessing exposures to mixtures subject to limits of detection. Epidemiology. 2010;21:S71–S76. doi:10.1097/EDE.0b013e3181cf0058.

2. Molitor J, Papathomas M, Jerrett M, Richardson S. Bayesian profile regression with an application to the National Survey of Children's Health. Biostatistics. 2010;11(3):484–498. doi:10.1093/biostatistics/kxq013.

3. Molitor J, Su JG, Molitor NT, Rubio VG, Richardson S, Hastie D, et al. Identifying vulnerable populations through an examination of the association between multipollutant profiles and poverty. Environmental Science and Technology. 2011;45(18):7754–7760. doi:10.1021/es104017x.

4. Liverani S, Lavigne A, Blangiardo M. Modelling collinear and spatially correlated data. Spatial and Spatio-temporal Epidemiology. 2016;18. doi:10.1016/j.sste.2016.04.003.

5. Liverani S, Hastie DI, Azizi L, Papathomas M, Richardson S. PReMiuM: An R Package for Profile Regression Mixture Models Using Dirichlet Processes. Journal of Statistical Software. 2015;64(7):1–30. doi:10.18637/jss.v064.i07.

6. Hoskovec L. mmpack: Implement methods for multipollutant mixtures analyses. R package version 0.1.0.; 2019. Available from: `https://github.com/lvhoskovec/mmpack`.

7. Dahl DB. Model-Based Clustering for Expression Data via a Dirichlet Process Mixture Model. Bayesian Inference for Gene Expression and Proteomics. 2006; p. 201–218. doi:10.1017/CBO9780511584589.011.

8. Bobb JF, Valeri L, Claus Henn B, Christiani DC, Wright RO, Mazumdar M, et al. Bayesian kernel machine regression for estimating the health effects of multi-pollutant mixtures. Biostatistics. 2015;16(3):493–508. doi:10.1093/biostatistics/kxu058.

9. Bobb JF, Henn BC, Valeri L, Coull BA. Statistical software for analyzing the health effects of multiple concurrent exposures via Bayesian kernel machine regression. Environmental Health: A Global Access Science Source. 2018; p. 1–10. doi:10.1186/s12940-018-0413-y.
